# Techno-economic analysis and environmental impact assessment of biodiesel production from bio-oil derived from microwave-assisted pyrolysis of pine sawdust

**DOI:** 10.1016/j.heliyon.2023.e22261

**Published:** 2023-11-11

**Authors:** Denzel Christopher Makepa, Chido Hermes Chihobo, Downmore Musademba

**Affiliations:** Department of Fuels and Energy Engineering, Chinhoyi University of Technology, Private Bag, 7724, Chinhoyi, Zimbabwe

**Keywords:** Microwave-assisted pyrolysis, Pine sawdust, Bio-oil esterification, Biodiesel, Techno-economic analysis

## Abstract

Pyrolysis stands out as a highly promising technology for converting biomass. Upgrading the bio-oil to meet the requirements for fuelling internal combustion engines is indispensable. This study evaluates the economic viability of microwave-assisted pyrolysis (MAP) of pine sawdust, followed by bio-oil esterification for the production of biodiesel. Aspen Plus® was used to simulate a facility that processed 2000 metric tonnes of pine sawdust per day. The minimum fuel selling price (MFSP) of biodiesel was established through the use of a discounted cash flow analysis. A life cycle assessment approach was used to evaluate the environmental impact assessment of biodiesel production. Process modelling findings revealed that the pyrolysis section yielded 65.8 wt% bio-oil, 8.9 wt% biochar, and 25.3 wt% NCGs. The biodiesel product yield was 48 wt% of the raw bio-oil, yielding 631.7 tonnes per day of biodiesel. With the cost of methanol playing a significant role, the overall capital investment was $286.1 MM and the total yearly operating expenses were $164.9 MM. The predicted MFSP for biodiesel is $2.31/L, with yearly operational expenses and biodiesel output being the most important factors. The emission from the biodiesel production process resulted in a global warming potential of 70.97 kg CO_2eq_. With an anticipated MFSP that is competitive with traditional diesel fuel, the study concludes that the method is economically viable. The results underline how crucial it is to optimize crucial process variables in order to increase the process's economic viability.

## 1 Introduction

Over the past few decades, the development of biofuels has drawn a lot of interest as a viable replacement for conventional fossil fuels. This is primarily because biofuels can potentially improve sustainability, minimize greenhouse gas emissions, and improve global energy security [1]. Worries over climate change and the environmental impact of non-renewable fuels have sparked interest in producing sustainable biofuels with a lower carbon footprint.

Pyrolysis is a practical method for transforming biodegradable materials into crude bio-oil that can be utilized in an integrated biorefinery to synthesize various biofuels and value-added chemicals. Pyrolysis, is the thermal degradation of carbonaceous biomass feedstocks in an inert environment, in this process complex organic molecules are broken down into less complicated ones [2]. The synthesis of bio-oil from biomass feedstocks can be optimized in yield and efficiency using microwave-assisted pyrolysis (MAP) [3,4]. Compared to conventional pyrolysis techniques, MAP can reduce processing times and boost bio-oil production because microwaves can quickly and evenly heat biomass feedstock [2]. While possessing comparable characteristics to petroleum-based diesel, bio-oil cannot be utilized directly as a fuel due to its instability and high acidity [5]. As a result, bio-oil requires upgrading to enhance its quality characteristics for use as fuel.

Bio-oil can be upgraded or transformed to biodiesel through esterification. Esterification is a process that involves reacting the bio-oil with an alcohol, such as methanol, to produce fatty acid methyl esters (FAMEs), respectively [6]. This process is similar to the transesterification process used to manufacture biodiesel from animal fat and vegetable oils. Bio-oil esterification can potentially reduce the acidity of bio-oil, improve its stability, and increase its energy density. The resulting FAMEs can be further purified for use or blended with conventional diesel.

While large-scale commercialization of biofuel production technologies utilizing bio-oil remains elusive, there is potential for biomass-based products to replace fossil fuel alternatives, provided they are produced sustainably and cost-effectively. One approach to assessing the economic feasibility of biomass conversion pathways is through techno-economic analysis (TEA) [3]. Several studies have utilized TEA to investigate the economics of various fast pyrolysis pathways. For example, Wright et al. [7] projected a product price of $2.11 and $3.09 per gallon of gasoline equivalent for a pyrolysis plant that converted biomass to diesel-range fuels and naphtha under hydrogen production and hydrogen purchase scenarios, respectively. Li et al. [8] projected MFSPs ranging between $1.11 and $1.13/L for pyrolysis of biomass for transportation fuel production, respectively. Brown et al. [9] assessed the techno-economic viability of various pathways that produced bio-chemicals through the integrated catalytic fast pyrolysis process, while Hu et al. [10] evaluated the economics of bio-chemicals, biofuels and hydrocarbon chemicals production through a fast pyrolysis bio-refinery. Hu et al. estimated minimum selling prices of $0.82/L for biofuels, $773.5 per metric ton for hydrocarbon chemicals and $433.7 per metric ton for bio-chemicals. These studies demonstrate the potential for cost-effective synthesis of biofuels and value-added products derived from biomass-through fast pyrolysis technology. However, challenges such as equipment costs, process optimization, and uncertainties in market demand and pricing must be addressed to achieve large-scale commercialization.

Most previous studies focused on traditional biofuel production systems [[10], [11], [12]] and have not explored the economics of upgrading bio-oil via the esterification route. A few studies have attempted to upgrade bio-oil via esterification [13,14], but information on the economics of this process is scarce. A thorough TEA and environmental impact assessment of bio-oil esterification for biodiesel synthesis can aid in the development of sustainable and affordable biodiesel production pathways. By conducting a thorough TEA of the bio-oil esterification process, the research adds to the existing knowledge base and provides valuable insights into the economic feasibility of this pathway. This information may be used to identify opportunities for cost reduction and process optimization, create novel catalysts and process conditions, and guide policy choices relating to the production of biofuels. Researchers, decision-makers, and industry stakeholders can collaborate to develop and promote the adoption of more sustainable and economical biodiesel production technologies, which can lessen reliance on non-renewable fossil fuels and slow down climate change, by utilizing the insights gained from TEA.

The central goal of the study was to assess the techno-economic feasibility of producing biodiesel using bio-oil derived from the MAP of pine sawdust. The study also sought to evaluate the sensitivity of the MFSP to these factors and to determine the critical process variables that influence the economic feasibility of the process.

## 2 Methods

### 2.1 Pine sawdust characterization

To ascertain the input characteristics of the biomass feedstock in Aspen Plus®, proximate and ultimate analysis of the pine sawdust was conducted. Samples of pine sawdust were taken at specified sawmills that only process pine (*Pinus Patula*) wood. The modified procedures outlined in ASTM D3173-5 were utilized to conduct the proximate analysis. For the elemental analysis, ASTM D5373 (2014) was followed, and the test was conducted using the Thermo Scientific Flash 2000 Organic Elemental Analyzer. Table 1 contains the necessary information for modelling pine sawdust in Aspen Plus®.

**Table 1:** The composition of pine sawdust on a dry basis.

Proximate analysis (wt.%)	
Fixed Carbon	14.24
Ash Content	0.28
Volatile Matter	78.19
Moisture Content	7.29
Elemental analysis (wt.%)	
C	51.60
N	0.04
H	5.20
O	43.16^a^

a Calculated from difference.

### 2.2 Process modelling

**Fig. 1:**
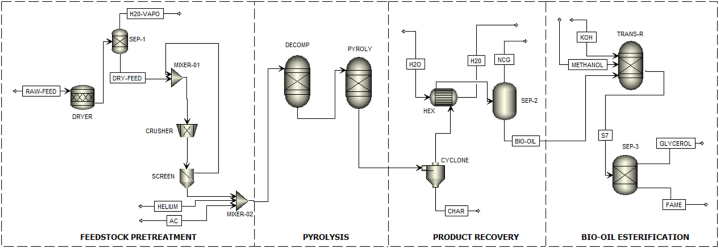
MAP flow diagram for the microwave-assisted pyrolysis of pine sawdust.

The MAP process of pine sawdust for bio-oil, char and syngas production, with subsequent bio-oil esterification to biodiesel was developed using Aspen Plus® V11 assuming a nth plant design. The model assumes the conversion of 2000 metric tonnes of pine sawdust on a daily basis. The model is comprised of four major processing steps: feedstock pre-processing, fast pyrolysis, pyrolysis product recovery and bio-oil esterification. A process flowsheet of the MAP process is presented in Fig. 1.

#### 2.2.1 Feedstock pre-processing

During feedstock pre-processing, the pine sawdust was sent to a drier operating at 125 °C and 1 atm to remove excess moisture. Effective drying of the feedstock is crucial in thermochemical processes as the presence of moisture in the feedstock consumes process heat resulting in decreased product yields. The drier was modelled in Aspen Plus® using an RSTOIC reactor. The drying process is necessary as low moisture content enhances the pyrolysis process and improves product quality [15]. The dried biomass was then sent to a crusher where the biomass particle size is reduced to approximately 2 mm and passes through a screen. It was essential to carry out this step to eliminate pine sawdust particles that were larger than the required size. The ground pine sawdust was sent to a feed/adsorbent mixer where it is mixed with activated carbon at a ratio of 1:50. Activated carbon is an effective microwave absorbent [16] and also catalyses the pyrolytic cracking of biomass.

#### 2.2.2 Fast pyrolysis

Fast pyrolysis is known as the thermochemical degradation of organic matter in an oxygen-deficient environment, at temperatures ∼500 °C, 1 atm pressure and short residence time ∼1s [17]. The microwave-assisted fast pyrolysis process was simulated using two sets of reactors in Aspen Plus®. A RYIELD reactor (DECOMP) was employed in modelling the decomposition of pine sawdust into conventional components, based on the ultimate and proximate analysis of the pine sawdust (Table 1), and a RGIBBS reactor (PYROLY) was employed in modelling the thermal conversion of biomass through MAP. The RGIBBS reactor was modelled at optimized conditions from a previous study (550 °C, 1 atm) [18] and the estimation of the product distribution is achieved through the minimization of Gibbs free energy. A constant flow of helium gas at 250 kmol/h through the reactor was used to provide an inert atmosphere to facilitate the pyrolysis process.

#### 2.2.3 Product recovery

The stream emanating from the PYROLY reactor is a combination of condensable vapours and non-condensable gases and entrained particles of ash and char. A high-volume cyclone operating at an efficiency of 0.9 was modelled to remove the entrained particles of ash and char from the process stream. To achieve high bio-oil quality and yields, it is important to rapidly condense the vapours immediately after they exit the PYROLY reactor. Secondary reactions that restricts the quantity and quality of the bio-oil collected are encouraged by prolonged residence times [7]. To achieve quick condensation of pyrolysis vapours, the vapour fraction is condensed using a heat exchanger that operates in counter-flow. The production of bio-oil was accomplished by condensing the vapours in indirect contact heat exchangers, and it can be stored safely at ambient conditions before further processing. The major constituents of non-condensable gases are carbon monoxide, hydrogen and light hydrocarbons. These can be burned to avail the heat essential for the drying process.

#### 2.2.4 Esterification

The bio-oil was esterified in a REQUIL reactor to produce FAMEs (biodiesel). The bio-oil was subjected to a reaction with excess methanol using a 1 wt% KOH catalyst, at a temperature of 65 °C and atmospheric pressure. Equations (1)–(5) were specified in Aspen Plus for the conversion of the fatty acids and carboxylic acids present in the bio-oil to biodiesel [19]. Glycerol was separated from the biodiesel as a by-product.

(1) $$\text{Oleic}\;\text{acid}\;\left(C_{18}H_{34}O_{2}\right)+\text{Methanol}\;\left({\text{CH}}_{3}\text{OH}\right)↔\text{Methyl}‐\text{Oleate}\left(C_{19}H_{36}O_{2}\right)+\text{water}\;\left(H_{2}O\right)$$

(2) $$\text{Palmitic}\;\text{acid}\;\left(C_{16}H_{32}O_{2}\right)+\text{Methanol}\;\left({\text{CH}}_{3}\text{OH}\right)↔\text{Methyl}‐\text{Palmitate}\left(C_{17}H_{34}O_{2}\right)+\text{water}\;\left(H_{2}O\right)$$

(3) $$\text{Linoleic}\;\text{acid}\;\left(C_{18}H_{32}O_{2}\right)+\text{Methanol}\;\left({\text{CH}}_{3}\text{OH}\right)↔\text{Methyl}‐\text{Linoleate}\left(C_{19}H_{34}O_{2}\right)+\text{water}\;\left(H_{2}O\right)$$

(4) $$\text{Acetic}\;\text{acid}\;\left(C_{2}H_{4}O_{2}\right)+\text{Methanol}\;\left({\text{CH}}_{3}\text{OH}\right)↔\text{Methyl}‐\text{Acetate}\left(C_{3}H_{6}O_{2}\right)+\text{water}\;\left(H_{2}O\right)$$

(5) $$\text{Formic}\;\text{acid}\;\left({\text{CH}}_{2}O_{2}\right)+\text{Methanol}\;\left({\text{CH}}_{3}\text{OH}\right)↔\text{Methyl}‐\text{Formate}\left(C_{2}H_{4}O_{2}\right)+\text{water}\;\left(H_{2}O\right)$$

### 2.3 Cost estimation

The Aspen Process Economic Analyzer (APEA) was utilized in estimating the expenses and size of equipment. The unit operations that were simulated in Aspen Plus were sent to APEA to approximate equipment procurement costs and conduct sizing measurements. The costs of the pyrolysis and esterification reactors were determined using the scaling equation (Equation (6)) as outlined by Shoaib Ahmed Khan et al. [20].

(6) $$C_{1}=C_{0}∙{\left(\frac{S_{1}}{S_{0}}\right)}^{0.6}$$

where C_1_ represents the new equipment cost, S_1_ represents the size of the new equipment, C_o_ represents the base equipment cost and S_o_ represents the base equipment size. To update the equipment cost to 2022, Equation (7) is applied, utilizing the Chemical Engineering Plant Cost Index (CEPCI).

(7) $$New\;equipment\;cost=Base\;equipment\;cost\times \frac{2022cost\;index\;value}{Base\;year\;cost\;index\;value}$$

The CEPCI value for 2022 is recorded as 699.0, while for 2018 it is 603.1 [21]. Table 2 presents the assumptions taken into account when calculating the total operating cost. Table 3 outlines the assumptions utilized in the discounted cash flow analysis.

**Table 2:** Operating cost parameters.

Material	
Biomass cost ($/ton)	80
Helium ($/ton)	12
Activated carbon ($/ton)	2000
KOH catalyst ($/ton)	2500
Methanol ($/ton)	600
**Utilities**	
Electricity ($/kWhr)	0.08
Cooling water ($/m^3^)	0.05

**Table 3:** Inputs for discounted cash flow analysis.

Economic inputs	
Income Tax	40 %
Revenue escalation	5 %
Required rate of return	10 %
Capital cost escalation	5 %
Operating cost escalation	3 %
Plant life	20 years

By summing up all the cost ratios, a Lang factor of 5.21 was attained, which represents the proportion of the total project investment (TPI) to the total procurement cost of equipment. This factor was utilized to determine the TPI. Total capital investment (TCI) was determined using the method as presented in Table 4 [3,22]. Estimations derived using this methodology usually possess an accuracy of approximately 70 % [7]. The capital cost associated with biomass pyrolysis and bio-oil esterification consists of direct and indirect costs, contingency, and location factors. The cost model incorporates regional labor, supervisor, and service costs. The Total Installed Cost (TIC) is estimated as 3.02 times the purchased equipment cost, encompassing installation expenses such as electrical wiring, plumbing, structures, and other related costs. Indirect costs encompass contractor's fees, supervision and technical costs, legal fees, and construction expenses. The rate is determined as 0.89 times the total purchased equipment cost. In determining the MFSP of biodiesel for a required rate of return of 10 % over 20 years, a modified DCFROR spreadsheet was utilized.

**Table 4:** Total project investment estimation method [3,22].

Parameter	Percent of delivered equipment cost (%)
Total Purchased Equipment Cost (TPEC)	100
Service facilities	55
Buildings (including services)	47
Electrical systems	31
Instrumentation and controls	26
Piping	10
Purchased equipment installation	39
Yard improvements	12
Total Installed Equipment Costs (TIEC)	TPEC * 3.20
Legal and contractors' fees	23
Engineering	32
Building	34
Contingency	15 % of Fixed capital investment (FCI)
TIC	1.26 * TPEC
FCI	TIEC + TIC
Working capital (WC)	75
TCI	FCI + WC

### 2.4 Sensitivity analysis

The analysis of MFSP's sensitivity was conducted as there were several assumptions made in the model that caused uncertainties in the analysis. By conducting a sensitivity analysis, it was possible to ascertain the factors that exert the greatest influence on the MFSP. The process involved assessing the MFSP by modifying a single factor while keeping all other factors constant. In this evaluation, the factors that were examined included fixed capital investment, interest rate, income tax rate, biodiesel yield, annual operating cost, biomass cost, methanol cost, catalyst cost, activated carbon cost, electricity cost, and utility water costs. In this study, the variation range was ±25 % of the base MFSP of biodiesel for the parameters evaluated, according to the most critical assumptions. The MFSP is assessed for the base case as well as for the low-end and high-end values of each parameter. The black bars represent the sensitivity of base MFSPs to a 25 % increase in the parameters, while the red bars illustrate the sensitivity of base MFSPs to a 25 % decrease in the parameters. In general, longer bars indicate higher sensitivity of base MFSPs to parameter changes, and vice versa.

### 2.5 Uncertainty analysis

The conclusions drawn from this research rely on a deterministic economic analysis that assumes accurate knowledge of all parameters. However, it is worthy noting that the costs and factors used to assess the profitability of the chemical process are subject to substantial volatility throughout the 20-year lifespan of the project. Although the sensitivity analysis varied only a single factor at a time, in reality, numerous factors would undergo simultaneous variations. Therefore, a Monte Carlo analysis was conducted on the biorefinery to assess the uncertainty of the process parameters. The triangular probability distribution was used with similar variations as those presumed in the MFSP sensitivity analysis. The analysis was carried out using Crystal Ball® software with 5000 trials, and the results were analysed using Microsoft Excel® software.

### 2.6 Environmental impact assessment

To evaluate the potential environmental impacts of biodiesel production, a lifecycle assessment (LCA) approach was employed. The LCA technique enables the assessment of environmental characteristics and potential consequences associated with a product, process, or service [23]. In this study, the entire lifecycle of the biofuel production technology utilizing MAP was evaluated using data obtained from Aspen Plus process simulation models and economic analyses.

A cradle-to-grave environmental impact assessment method was adopted, considering the complete lifecycle of the biodiesel production process. The functional unit chosen as a reference for this research was 1 tonne of biodiesel produced. The LCA was conducted using openLCA 2.0 software and the Ecoinvent database, which facilitated the assessment of the global warming potential based on the ReCiPe 2016 (H) midpoint impact assessment method.

Specifically, the input and output data for pine cultivation and timber production were sourced from a forestry and sawmilling company located in the Eastern Highlands of Zimbabwe. This data formed the basis for evaluating the environmental impacts associated with these stages of the biofuel production process. To estimate the mass and energy balances related to the biomass pyrolysis and bio-oil esterification system, Aspen Plus® v11 software was utilized. This enabled a comprehensive analysis of the environmental implications associated with these specific processes. Table 5 presents a detailed lifecycle inventory of producing biodiesel from bio-oil derived from MAP of pine sawdust.

**Table 5:** Lifecycle inventory for producing 1 ton of biodiesel.

Flow	Unit	Amount
*Inputs*		
Ammonium nitrate phosphate	kg	50
Ammonium nitrate phosphate	kg	2250
Charcoal	kg	63.2
Diesel	kg	320
Electricity	kWh	575
Helium	kg	12
Methanol	kg	3000
Potassium chloride	kg	190
Sodium hydroxide	kg	10
Wood sawdust	kg	3160
*Outputs*		
Acetic acid	kg	0.92
Biodiesel	kg	1000
Carbon dioxide	kg	1170
Carbon monoxide	kg	100
Charcoal	kg	860
Copper	kg	16
Dimethoate	kg	4.4
Dinitrogen monoxide	kg	0.012
Ethane	kg	0.42
Formic acid	kg	1.69
Furfural	kg	0.62
Glycerol	kg	300
Hydrogen	kg	2
Iron	kg	125000
Magnesium	kg	26630
Manganese	kg	890
Methane	kg	15.54
Nitrogen dioxide	kg	0.26
Nitrogen monoxide	kg	3.2
Particulates, <10 um	kg	0.11
Potassium	kg	30200
Propionic acid	kg	4.6
Propylbenzene	kg	0.15

## 3 Results and discussions

### 3.1 Process modelling results

The model processed 2000 dry metric tonnes per day (MTPD) of pine sawdust. The process modelling results showed that the pyrolysis section yielded 65.8 wt% bio-oil, 8.9 wt% biochar and 25.3 wt% NCGs. The results are consistent with the process modelling results of previously published works [10,18,24]. The bio-oil was further upgraded via esterification to produce biodiesel. The biodiesel product yield was 48 wt% of the raw bio-oil. The final product yields of the process are presented in Table 6. The produced biofuel has several applications as a transportation fuel for diesel engines, boiler fuel for electricity generation in power plants through steam production, feedstock for chemical production and a potential sustainable aviation fuel [25,26]. The process produced a by-product stream, also known as glycerol. Glycerol possesses significant value as a versatile chemical, finding applications in various sectors including soap manufacturing, cosmetics, and as a food additive. The glycerol produced during the esterification of bio-oil can be sold as a separate product, providing an additional source of revenue [27].

**Table 6:** Process yields of the microwave-assisted pyrolysis of pine sawdust.

Input	Output	Yield (MTPD)
Biomass (2000 MTPD)	Bio-oil (65.8 wt%)	1316.0
	Biochar (8.9 wt%)	178.0
	NCGs (25.3 wt%)	506.0
Bio-oil (1316.0 MTPD)	Biodiesel (48.0 wt% of bio-oil)	631.7
	By-products (52.0 wt% of bio-oil)	684.3

### 3.2 Economic analysis

**Fig. 2:**
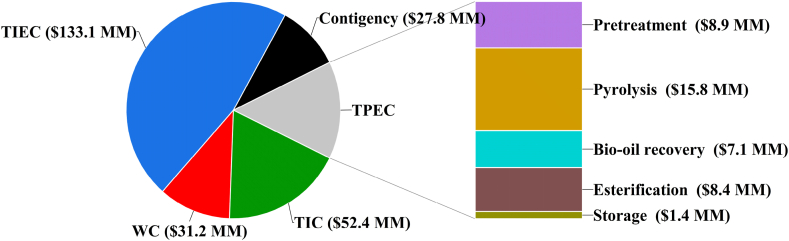
TCI for the production of biodiesel from pine sawdust.

The TCI is visualized in Fig. 2. It represents the cumulative total of the TPEC, TIEC, TIC, WC and project contingency of $41.6 million (MM), $133.1 MM, $52.4 MM, $31.2 MM and $27.8 MM, respectively (Table 7). The stacked bar chart in Fig. 2 presents an analysis of the total purchased equipment costs for different areas. The pyrolysis section accounts for the largest portion of the total installed equipment cost, representing approximately 37.9 % or $15.8 MM. The biomass pretreatment section also incurs significant installed equipment costs, contributing 21.4 % of the Total Purchased Equipment Cost (TPEC) with a value of $8.9 MM. The expenses for pretreatment equipment are primarily influenced by the inclusion of a dryer and either a ball mill or biomass crusher. The bio-oil esterification section contributed 20.3 % to the TPEC, with a value of $8.4 MM. This cost is influenced by the utilization of an esterification reactor which converts the bio-oil fraction to biodiesel and the final product separator which separates the biodiesel from glycerol. The pyrolysis product recovery section also contributed a significant amount to the TPEC. The cost was driven by the employment of condensate separators, heat exchangers and cyclone separators, contributing 17.1 % to the TPEC with a value of $8.4 MM. The biodiesel storage facilities contributed 3.3 % of the TPEC with a value of $1.4 MM.

**Table 7:** Summary of economic evaluation of biodiesel production from bio-oil derived from MAP of pine sawdust.

Cost evaluation parameter	Value
Total capital investment	$286.1 MM
Total purchased equipment costs	$41.6 MM
Total installed equipment costs	$133.1 MM
Total annual operating costs	$164.9 MM
Minimum Fuel selling price	$2.31/litre

**Fig. 3:**
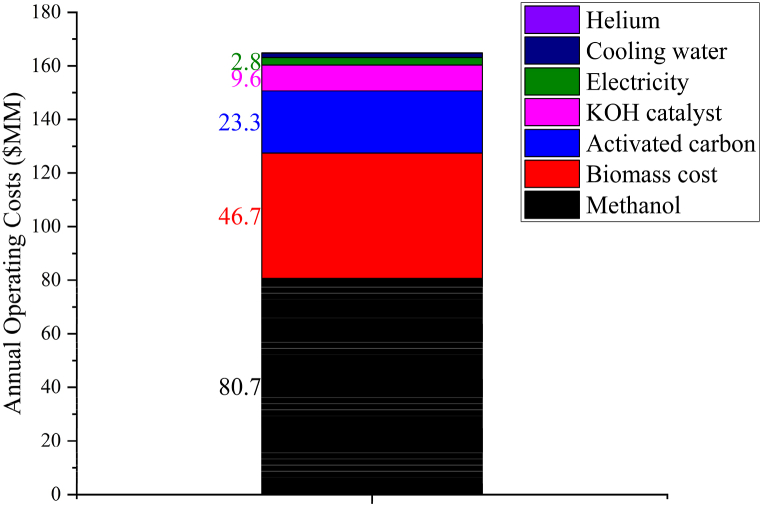
Total annual operating costs of biodiesel production from pine sawdust.

The annual total operating costs amount to $164.9 MM, as shown in Fig. 3. Among these costs, the expense associated with methanol constitutes the largest portion, amounting to $80.7 MM per year, followed by biomass cost ($46.7 MM), activated carbon ($23.3 MM), and catalysts ($9.6 MM). Utilities contributed $4.5 MM to the annual operating cost with helium gas contributing the least with a value of $0.1 MM. These findings align with similar techno-economic analyses [7,24,28], that highlight feedstock and consumables costs as the primary components of annual operating expenses for fast pyrolysis facilities. To comply with the 10 % required rate of return, a MFSP of $2.31/litre of biodiesel was estimated from the DCFROR analysis (Table 7). This price is significantly higher than the price of conventional diesel in Zimbabwe ($1.65/litre) [29]. One possible reason for this price difference is the costly nature of establishing and maintaining the necessary equipment and infrastructure for biodiesel production. As with previous TEAs, product yields, fixed capital costs, biomass and other consumables costs have the greatest impacts on the MFSP [8,28]. A previous study estimated a MFSP of $1.38/litre for biodiesel obtained from sugarcane lipids [30]. The differences might be due to the complex thermochemical conversion of biomass, which requires expensive processing equipment. A study by Kedia et al. [31] estimated a MFSP of biodiesel obtained from non-edible oils of $2.15/litre, which was close to the MFSP estimated in this study.

### 3.3 Sensitivity analysis

**Fig. 4:**
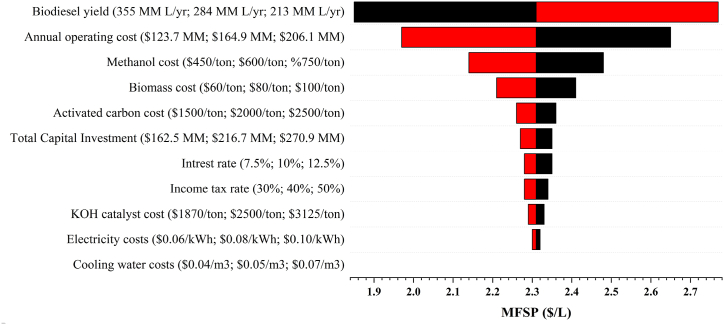
Sensitivity analysis of the MFSP of biodiesel produced from pine sawdust.

Fig. 4 presents the sensitivity analysis outcomes of biodiesel production from pine sawdust. The biodiesel yield has the highest sensitivity on the MFSP. A 25 % increament in the yield of biodiesel resulted in a 20 % decrease in the MFSP of biodiesel to $1.85/L. Conversely, a 25 % decrease in the biodiesel yield resulted in a 20 % increase in the MFSP to $2.77/L. Similar trends were observed in previous studies [[32], [33], [34]], which employed the fast pyrolysis technique for biofuel production. This implies that improving the process operating conditions to obtain more biodiesel can significantly reduce the MFSP. Another key parameter which affects the sensitivity of the MFSP is the annual operating cost. A 25 % increament in the annual operating cost increases the biodiesel yield by 15 % to $2.65/L, and reducing the annual operating costs reduces the biodiesel yield by 15 % to $1.97/L. The process inputs which greatly affect the sensitivity of the MFSP are the costs of methanol, biomass and activated carbon. A 25 % increase in the methanol, biomass and activated carbon increased the MFSP from $2.31/L (base case) to $2.48/L, $2.41/L and $2.36/L, respectively. Conversely, a 25 % decrease in the methanol, biomass and activated carbon reduced the MFSP to $2.14/L, $2.21/L and $2.26/L, respectively. Among the process inputs, catalyst costs and electricity costs had the least sensitivity to the MFSP, and the effect of variation in the cost of utility water was negligible. The TCI also has a considerable effect on MFSP. The interest rate and the income tax rate also affected the sensitivity of the MFSP by a margin of 1.3 %.

### 3.4 Uncertainty analysis

To assess the uncertainty of the TEA results, a Monte Carlo analysis was performed to determine the distribution of the MFSP. The sensitivity analysis has shown that the biodiesel yield, annual operating costs, methanol cost, biomass cost, activated carbon costs, and TCI have the most significant impact on the MFSP. Therefore, these six model parameters were regarded as fluctuating variables in the Monte Carlo analysis, with a varied range of ±25 % as in the sensitivity analysis. During the simulation, 5000 random MFSPs were generated.

**Fig. 5:**
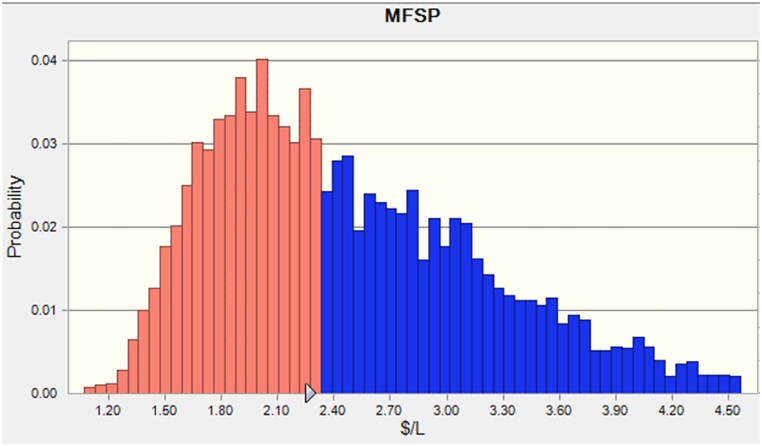
Probability distribution of MFSP for biodiesel.

**Fig. 6:**
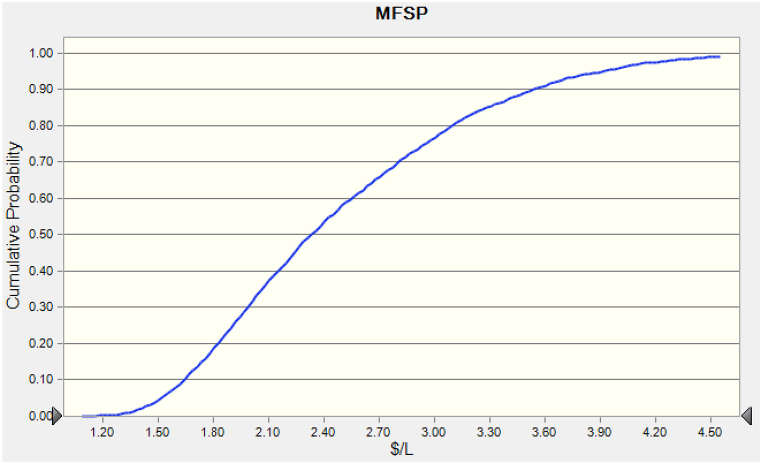
Cumulative probability of the MFSP of biodiesel.

The probability distribution of MFSP for biodiesel is illustrated in Fig. 5. An average MFSP for biodiesel of $2.49/L with a standard deviation of 0.74 % was observed from 5000 runs. This means that the biodiesel needs to be sold at $2.49/L or higher to cover the production costs. This is an important metric for the profitability of the biorefinery and indicates the competitiveness of biodiesel in the market. The standard deviation of 0.74 % indicates that the MFSP values have low variance, which means that the biodiesel production cost is relatively consistent across the 5000 simulation runs. This suggests that the production process is stable and well-controlled and that the biorefinery can produce biodiesel with a consistent cost structure. Fig. 6 presents the collective probability of the resultant MFSP, which ranged from $1.10 to $4.50/L with an 80 % chance of being within the range of $1.30 to $3.10/L. MFSP for the biorefinery has a 50 % chance of being less than $3.21/L.

### 3.5 Environmental impact assessment results

A GWP of 70.97 kg CO_2eq._/ton of biodiesel produced was estimated in this study. The value indicates the emissions of greenhouse gases linked with the evaluated biodiesel production process. When comparing this GWP value with findings from other studies, it is evident that the assessed biodiesel production method using MAP demonstrates relatively lower emissions in terms of global warming potential. The lower value could be attributed to the use of biomass as a feedstock for biodiesel production can result in carbon sequestration, as the growing plants absorb atmospheric CO_2_ during their lifecycle. This carbon uptake can offset or even exceed the emissions generated during the biofuel production process, leading to a net reduction in CO_2_ equivalents.

Dufour & Iribarren [35] conducted a study on the production of biodiesel from animal waste and used cooking oil and reported a GWP value of 652.16 kg CO_2eq._/ton of biodiesel produced. Carvalho et al. [36] evaluated the production of biodiesel from solaris seed tobacco and obtained a GWP value of 665.00 kg CO_2eq._/ton of biodiesel produced. Al-Mawali et al. [37] assessed the production of biodiesel from waste date seed oil and obtained a GWP value of 1114.25 kg CO_2eq._/ton of biodiesel produced. Comparing these values, it is evident that the biodiesel production process evaluated in this study using MAP technology exhibits a relatively lower GWP than the other assessed methods. This suggests that the evaluated process may have a lesser impact on climate change in terms of greenhouse gas emissions per ton of biodiesel produced.

The variation in GWP values among these studies can be attributed to several factors. First, the choice of feedstock plays a significant role in determining the emissions associated with biodiesel production. Different feedstocks have varying carbon contents and cultivation processes, leading to variations in GWP values. Additionally, variations in the production technologies, process efficiencies, and energy sources utilized can contribute to differences in emissions. The specific system boundaries, allocation methods, and data quality employed in each study can also influence the calculated GWP values.

## 4 Assumptions and limitations of the study

Economic analysis often involves assumptions about investment costs, operating expenses, and market prices for feedstock, biodiesel, and co-products. Fluctuations in these factors can significantly impact the feasibility and profitability of biodiesel production. The study may assume certain economic conditions that may not accurately reflect real-world scenarios. Assumptions have been made about the availability and sustainability of the resources required for the entire biodiesel production process, including feedstock, chemicals, and catalysts. The study's findings and conclusions may be specific to the geographical location or context in which the research was conducted. Factors such as climate, infrastructure, policy frameworks, and market conditions can significantly influence the techno-economic and environmental performance of biodiesel production.

## 5 Conclusions

The pyrolysis section yielded 65.8 wt% bio-oil, 8.9 wt% biochar, and 25.3 wt% NCGs. The biodiesel product yield was 48 wt% of the raw bio-oil, yielding 631.7 tons/day of biodiesel. The TCI was $286.1 MM, which is the sum of the TPEC, TIEC, TIC, WC and project contingency of $41.6 MM, $133.1 MM, $52.4 MM, $31.2 MM and $27.8 MM, respectively. Methanol costs accounted for the majority of the annual operational costs ($164.9 MM). A MFSP of $2.31/L of biodiesel was estimated. The emissions of greenhouse gas associated with the biodiesel production process resulted in a GWP of 70.97 kg CO_2eq._ Furthermore, future research could explore the socio-economic implications of this study, including assessing the impact on rural communities, job creation potential, economic viability for small-scale enterprises, and the social acceptance and adoption of this technology. Investigating these aspects would provide valuable insights into the broader implications and feasibility of implementing this process.

## Data availability statement

No data was used for the research described in the article.

## CRediT authorship contribution statement

**Denzel Christopher Makepa:** Writing – original draft, Validation, Methodology, Investigation, Formal analysis, Conceptualization. **Chido Hermes Chihobo:** Writing – review & editing, Supervision, Formal analysis, Conceptualization. **Downmore Musademba:** Validation, Supervision, Investigation, Formal analysis.

## Declaration of competing interest

The authors declare that they have no known competing financial interests or personal relationships that could have appeared to influence the work reported in this paper.
